# The european primary care monitor: structure, process and outcome indicators

**DOI:** 10.1186/1471-2296-11-81

**Published:** 2010-10-27

**Authors:** Dionne S Kringos, Wienke GW Boerma, Yann Bourgueil, Thomas Cartier, Toralf Hasvold, Allen Hutchinson, Margus Lember, Marek Oleszczyk, Danica Rotar Pavlic, Igor Svab, Paolo Tedeschi, Andrew Wilson, Adam Windak, Toni Dedeu, Stefan Wilm

**Affiliations:** 1 NIVEL, Netherlands Institute for Health Services Research, Otterstraat 114-118, 3500 BN Utrecht, Netherlands; 2 Institute for Research and Information in Health Economics IRDES, 10 rue Vauvenargues, 75018 Paris, France; 3 University of Tromsø, Department of Community Medicine ISM, Department of Community Medicine, Faculty of Health Sciences, University of Tromsø, 9037 Tromsø, Norway; 4 University of Sheffield, School of Health and Related Research ScHARR, Regent Court 30, Regent Street, S1 4DA Sheffield, UK; 5 University of Tartu, Department of Polyclinical and Family Medicine, Ülikooli 18, 50090 Tartu, Estonia; 6 Jagiellonian University Medical College, Department of Family Medicine, Bochenska 4, 31-061 Krakow, Poland; 7 University of Ljubljana, Department of Family Medicine, Vrazov trg 2, 1000 Ljubljana, Slovenia; 8 Bocconi University, Centre for Research on Health and Social Care Management CERGAS, Via Sarfatti 25, 20135 Milan, Italy; 9 University of Leicester, Department of Health Sciences, 22-28 Princess Road West, LE1 6TP Leicester, UK; 10 Primary Care Research Institute IDIAP Jordi Gol, Av. Gran Via de les Corts Catalanes, 587 Àtic, 08007 Barcelona, CIF G-60954104, Spain; 11 Private University Witten/Herdecke gGmbH, Institute of General Practice and Family Medicine, Alfred-Herrhausen-Straße 50 D - 58448 Witten, Germany

## Abstract

**Background:**

Scientific research has provided evidence on benefits of well developed primary care systems. The relevance of some of this research for the European situation is limited.

There is currently a lack of up to date comprehensive and comparable information on variation in development of primary care, and a lack of knowledge of structures and strategies conducive to strengthening primary care in Europe. The EC funded project Primary Health Care Activity Monitor for Europe (PHAMEU) aims to fill this gap by developing a Primary Care Monitoring System (PC Monitor) for application in 31 European countries. This article describes the development of the indicators of the PC Monitor, which will make it possible to create an alternative model for holistic analyses of primary care.

**Methods:**

A systematic review of the primary care literature published between 2003 and July 2008 was carried out. This resulted in an overview of: (1) the dimensions of primary care and their relevance to outcomes at (primary) health system level; (2) essential features per dimension; (3) applied indicators to measure the features of primary care dimensions. The indicators were evaluated by the project team against criteria of relevance, precision, flexibility, and discriminating power. The resulting indicator set was evaluated on its suitability for Europe-wide comparison of primary care systems by a panel of primary care experts from various European countries (representing a variety of primary care systems).

**Results:**

The developed PC Monitor approaches primary care in Europe as a multidimensional concept. It describes the key dimensions of primary care systems at three levels: structure, process, and outcome level. On structure level, it includes indicators for governance, economic conditions, and workforce development. On process level, indicators describe access, comprehensiveness, continuity, and coordination of primary care services. On outcome level, indicators reflect the quality, and efficiency of primary care.

**Conclusions:**

A standardized instrument for describing and comparing primary care systems has been developed based on scientific evidence and consensus among an international panel of experts, which will be tested to all configurations of primary care in Europe, intended for producing comparable information. Widespread use of the instrument has the potential to improve the understanding of primary care delivery in different national contexts and thus to create opportunities for better decision making.

## Background

### A need for up-to-date comparable primary care information

Primary care is the first level of professional care in Europe where people present their health problems and where the majority of the population's curative and preventive health needs are satisfied. Therefore primary care services should be available close to where people are living with no obstacles to access. Primary care is generalist care, focused on the person with a felt health problem in his or her social context, rather than on the optional diseases. The mix of disciplines which make up the primary care workforce may differ from country to country, but general practice or family practice is often considered as the core of primary care. Besides family practitioners, the most common primary care providers in Europe are general internists, general paediatricians, pharmacists, primary care nurses, physiotherapists, speech therapists, and mental health care workers [1,2].

Scientific research, both international comparative and within the United States, has provided evidence on benefits of well developed primary care systems, in terms of better coordination and continuity of care and better opportunities to control costs [2-7]. However, since the relevance of some of this research for the European situation is limited, more in-depth analyses are needed to corroborate these findings. The variety of models of organisation and provision of health care services found in Europe, are favourable circumstances to undertake sound and comprehensive studies on the merits of primary care for health care systems in general [8]. The rich diversity of regulatory mechanisms, funding schemes and modes of financial and non-financial incentives for providers as well as users of services makes Europe a laboratory for comparative research and a pool of good practices [9].

Getting insight in variation and effect of elements of primary care is not an academic exercise. The WHO World Health Report 2008, titled 'Primary health care now more than ever', has clearly articulated the need to mobilize the production of knowledge on primary care [10]. Despite the broad agreement about the merits of well organised primary care systems, current knowledge about its active ingredients is inconclusive. Better international comparative data and analyses of good practices will produce information to policy makers and those responsible for provision of services about the drivers of strong primary care [10-13]. Health reforms in many European countries share the aim to further develop the first level of care, and as a result there is a demand for comparative information and a growing tendency to learn from foreign experiences [14-17].

### An instrument for a multidimensional system

Primary care is a multi-dimensional (sub)system in which structural elements should facilitate access and utilisation of a range of coordinated services that aim to contribute to a population's health. The structural elements consist of regulation, economic conditions and human and material resources. The services provided together form the care process. Better health is a major outcome of the system but efficiency and equity are also considered as such. In a recent review of the literature on primary care, we identified ten dimensions, including governance; economic conditions; workforce development; access to services; continuity of care; coordination of care; comprehensiveness of care; quality of care; efficiency of care; and equity in health [8]. Each dimension was further broken down to a number of key attributes, which were called features (see Table 1).

**Table 1:** Result from the systematic literature review: identified primary care dimensions and features

PC Dimension	Feature
Governance of the PC system	1. Health (care) goals; 2. Policy on equity in access; 3. (De)centralization of PC management and service development; 4. Quality management infrastructure; 5. Appropriate technology in PC; 6. Patient advocacy; 7. Ownership of PC practices; 8. Integration of PC in the health care system.
Economic conditions of the PC system	1. Health care expenditure; 2. PC expenditures; 3. Health care funding system; 4. Employment status of PC workforce; 5. Remuneration system of PC workforces; 6. Income of PC workforce.
PC workforce development	1. Profile of PC workforce; 2. Recognition and responsibilities of PC disciplines; 3. Education and retention; 4 Professional associations; 5. Academic status of PC disciplines; 6. Future development of PC workforce.
Access to PC services	1. Availability of PC services; 2. Geographic access of PC services; 3. Accommodation of accessibility (incl. physical access); 4. Affordability of PC services; 5. Acceptability of PC; 6. Utilisation of PC services; 7. Equality in access.
Continuity of care	1. Longitudinal continuity of care; 2. Informational continuity of care; 3. Relational continuity of care; 4. Management continuity of care.
Coordination of care	1. Gatekeeping system; 2. PC practice and team structure; 3. Skill-mix in PC; 4. Integration of PC-secondary care; 5. Integration of PC and public health.
Comprehensiveness of PC	1. Medical equipment available; 2. First contact for common health problems; 3. Treatment and follow-up of diseases; 4. Medical technical procedures and preventive care; 5. Mother/child/reproductive health care; 6. Health promotion.
Quality of PC	1. Prescribing behaviour of PC providers; 2. Quality of diagnosis and treatment in PC; 3. Quality of chronic disease management; 4. Quality of mental health care; 5. Quality of maternal and child health care; 6. Quality of health promotion; 7. Quality of preventive care; 8. Effectiveness; 9. Practice safety.
Efficiency of PC	1. Allocative and productive efficiency; 2. Technical efficiency; 3. Efficiency in performance of PC workforce.
Equity in health	1. Equity in health

#### Objective

This article aims to describe the development of measurable indicators on the basis of characteristics (called dimensions and features) of primary care systems identified in the literature. This set of indicators and its underlying structure of dimensions and features will be referred to as the Primary Care Monitoring System (in short: PC Monitor). The PC Monitor is meant to produce comparable information of the variation of primary care systems across Europe. The study is part of the EC funded project Primary Health Care Activity Monitor for Europe (PHAMEU), that aims to describe and compare primary care systems in 31 European countries [18].

## Methods

The PC Monitor is developed in four steps: (1) a systematic literature review to identify dimensions and features of primary care; (2) development of indicators on the basis of results of the systematic literature review; (3) an evaluation among primary care experts of these indicators; and (4) testing the feasibility of the PC Monitor by implementing it in 31 European countries. This paper focuses on the first three steps. The results of step 4 will be described in a separate paper.

### Systematic literature review

**Figure 1:**
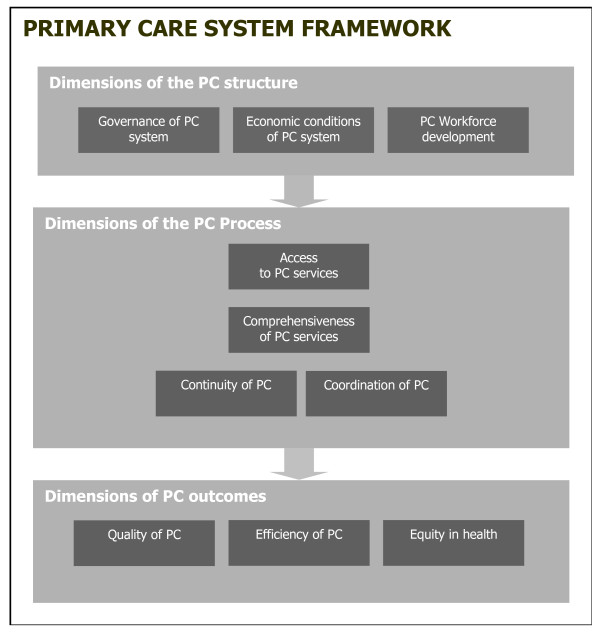
**Primary Care System Framework**.

A systematic literature review on original research and systematic reviews published between 2003 and July 2008 has been the basis of this study [8]. For practical reasons, such as time and financial constraints, the review was limited to this 5 year period. This review used a framework for primary care consisting of three levels: structure, process and outcome (see Figure 1); inspired by Donabedian's health system analysis approach [19]. Previous studies have shown the suitability of this approach for primary care systems [20-22].

The *structure *of a primary care system consisted of three dimensions: 1) governance; 2) economic conditions; and 3) workforce development. Four dimensions were related to the primary care *process*: 4) access; 5) continuity of care; 6) coordination of care; and 7) comprehensiveness of care. Three dimensions applied to its *outcome*: 8) quality of care; 9) efficiency of care; and 10) equity in health.

Subsequently each of the dimensions was detailed in specific features, which have been listed in Table 1. The strategy and results of the systematic literature review have been published elsewhere [8].

### Development of indicators

To work out the features identified in the systematic literature review measurable indicators were collected in a provisional list as follows. Firstly, the publications included in the literature review were searched for measurable descriptions. Secondly, a number of international databases (OECD Health Data, WHO Health for All Database, Eurostat, World Bank HNPStat's, EUPHIX) were searched for 'ready-made' indicators. Where these were not available, the research team developed measurable indicators.

In a first elimination round the researchers evaluated the indicators on the provisional list against four criteria:

• *Relevance*: covering an essential aspect of a dimension;

• *Precision: *precise formulation assuring easy-to-fill data (preferably numerical);

• *Flexibility: *likely to fit in various health systems in Europe;

• *Discriminating power: *yielding a range and variety of possible answers.

As it turned out that some indicators were specifications of other, more general indicators, in the long list that resulted from this first elimination a hierarchy was made in core indicators and indicators addressing additional information items.

### Further reduction of the long list of indicators

The long list of indicators was evaluated by the authors and eight other experts from various countries (including academics in family medicine, family practitioners and health services researchers). The aim was to arrive at a feasible and balanced set of essential indicators. The evaluators were asked to examine each indicator (and its additional information item) for its suitability to describe and compare primary care systems across countries. Indicators and items were scored on a four-point scale, ranging from zero ('not useful for primary care system comparison') to four ('essential for primary care system comparison'). In addition, they were asked to comment on the indicators (in terms of the criteria) and to provide possible suggestions for improvement. For each indicator the average score of the expert evaluation was calculated and this score was used in a procedure to reduce the long list. The following criteria were applied:

• A written comment by any evaluator that the indicator should be excluded by lack of compliance to criteria resulted in exclusion;

• Indicators more than 0.5 points below the average score of all indicators of that dimension were excluded;

• If there were more than 10 indicators on a feature, only 10 with the highest scores were included.

Evaluators could suggest to rephrase indicators or to include new ones. These were subjected to a consensus procedure during a meeting with all evaluators.

## Results

### Evaluation of provisional indicators

**Figure 2:**
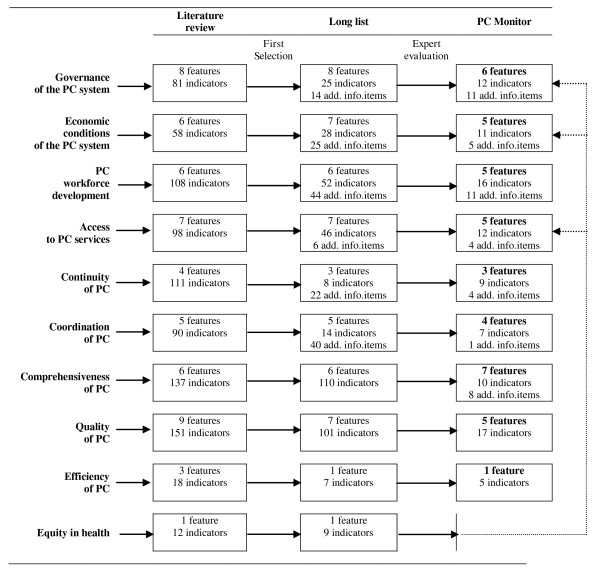
**Successive steps in the development of features and indicators for the PC Monitor**.

The selection process from the literature review via the long list of indicators to the final set of the PC Monitor has been summarised in figure 2.

On the basis of the systematic literature review (which included 85 publications) a provisional set of 55 features and 864 provisional indicators were collected. After the first elimination round 51 features, 400 indicators and 151 additional information items were left which were subsequently screened by the authors and eight other evaluators. This resulted in the final set of 41 features, 99 indicators and 44 additional information items, which together make the PC Monitor. No separate feature on equity remained, however, a number of indicators of governance, economic conditions and access also covered equity.

Table 2 provides an impression of the selection process by showing, for each of the 10 dimensions the three indicators with the highest scores and the three indicators with the lowest scores (and which, subsequently, were removed).

**Table 2:** Evaluation of suitability of long list indicators; selected results

Dimension		Results of evaluation: selected indicators with the highest (H) and lowest (L) average score*
Governance of the PC system	H	Is (near) universal financial coverage for PC services guaranteed by a publicly accountable body (government, or government-regulated insurer)? (3.42); Has a national primary care policy been formulated? (3.30); Is a national survey system or surveillance systems in place for monitoring the performance of the PC system (e.g. morbidity, mortality and process features)? (3.21)
	L	Provide a summary of the content of national standards on PC service delivery that allow PC practices to develop differently in their services delivery (1.63); Tasks and professionals included in legislation on possibilities of task substitution or delegation in PC (2.00); PC-oriented patient organisations currently being active (name, purpose, and number of members) (2.01)
Economic conditions of the PC system	H	Payment methods used for general practitioners?(Fee-for-service; Capitation payment; Salary; Mixed) (3.58); % of population covered for out-patient medical care by soc. health insurance (3.40); Method of health care financing for majority of (3.16)
	L	Public expenditure on dental services as % of GDP (1.42); Private expenditure on dental services as % of GDP (1.50); Public expenditure on over-the-counter medicines as % of GDP (1.68)
PC workforce development	H	Vocational training for general practice/family medicine in place? (3.55); Status of vocational training for general practice/family medicine (obligatory or voluntary) (3.57); Total nr. of active GPs as a ratio to total nr. of active specialists (3.39)
	L	% of (re)trained PC professionals (other than general practitioners, physiotherapists, pharmacists, dentists or midwives) active in their profession of training (1.26); Total number of posts of PC professionals (other than the previously listed PC professions) currently vacant per 1000 inhabitants (1.42); % of active female PC professionals (other than the previously listed PC professions) (1.49)
Access to PC services	H	Number of general practitioners per 100,000 population (3.74); Number of PC nurses per 100,000 population (3.56); Number of general practice consultations per capita per year (3.32)
	L	Differences in dentist visits by income quintile (or education) (1.73); Number of consultations with PC professionals (other than general practitioners, physiotherapists, pharmacists, dentists, midwives) per capita per year (1.76); Differences in physiotherapy visits by income quintile (or education) (1.86)
Continuity of care	H	Population/patients registered with a general practitioner (3.51); Average PC practice list size (3.45); Items normally recorded in patients' medical file for every encounter (reason of visit; problem and/or diagnosis; supporting data; treatment plan; medication details) (3.43)
	L	Usual Provider Continuity Index: proportion of visits to one's own PC physician relative to the total nr. of visits to all physicians in the past year (1.91); Average length of PC provider-patient relationship (2.08); Average practice list turnover: Nr. of new patients in a period divided by the nr. of registered patients at the end of the period (2.16)
Coordination of care	H	Patients having the possibility to directly access hospital based specialists (3.62); Patients having possibility to directly access emergency departments? (3.54); Patients having the possibility to directly access general practitioners? (3.49)
	L	Predominant PC-Public Health Collaboration models in place (1.85); Specialist outreach models available for specific (chronic) conditions (2.18); If no direct access to speech therapists, can these be consulted if paid out of pocket (2.21)
Comprehensiveness of PC	H	(Estimated) % of PC facilities usually carrying out immunizations for flu or tetanus (3.15); (Est.) % of PC providers usually providing first contact care to a man aged 28 with a first convulsion (3.09); (Est.) % of PC facilities usually involved in influenza vaccination for high-risk groups (3.08)
	L	(Est.) % of PC providers that regularly pay attention to social services (1.81); (Est.) % of PC facilities involved in blood typing and antibody screening for prenatal patients (1.90); (Est.) % of PC facilities involved in school health care (1.92)
Quality of PC	H	% of infants vaccinated against hepatitis B (2.99); % of infants vaccinated against invasive disease due to Haemophilius influenza type b (2.99); % of women aged 21-64 yrs who had at least 1 Pap test in the past 3 yrs (2.99)
	L	Mortality for persons with severe psychiatric disorders per 100,000 (1.24); % of pregnant women having received a hepatitis B screening during their pregnancy (1.28); Potential life years lost of premature mortality from bronchitis (1.35)
Efficiency of PC	H	Number of GP consultations per capita per year (3.34); Average consultation length (in minutes) of GPs (2.83); Number of new referrals from GPs to medical specialists per 1000 listed patients per year (2.82)
	L	Nr. of GP consultations in the surgery as % of all GP-patient contacts (2.24); Nr. of home visits as % of all GP-patient contacts (2.63); Nr. of telephone consultations as % of all GP-patient contacts (2.72)
Equity in health	H	Relative inequality (ratio between the rate of mortality in lowest and highest educational group) for avoidable mortality (2.34); Relative inequality for cardio-respiratory conditions (2.29); Relative inequality for mortality of infectious diseases (2.17)
	L	Relative inequality for mortality of tuberculosis (1.73); same for pneumonia and influenza (1.73); same for asthma (1.92)

* Judgement of evaluators: 0 = 'not useful', 1 = 'less important', 2 = 'important', 3 = 'very important', 4 = 'essential for PC comparison'.

In diminishing order, indicators/items for continuity, coordination, efficiency, comprehensiveness, accessibility, and governance were rated as very important. Indicators for economic conditions, workforce development and quality of care received a somewhat lower rating. The answers among evaluators were most similar for the indicators of the equity dimension, which received the lowest average score.

#### Equity in health

Equity in health is the absence of systematic and potentially remediable differences in health status across population groups [8]. Indicators on the equity dimension were relatively scarce and all received very low scores in the evaluation. Suitability of the equity in health indicators was rated low because they were not or just partially amenable to primary care (for example, equality in mortality of infectious diseases). With indicators on disparities in health the major difficulty was that they were influenced by various other factors than disparities in (primary) health care access and use; also social conditions in which people live and work played a role [23]. As a consequence, equity in health was not included in a monitor dealing with primary care. This does not mean, however, that equity in health, as an important health system outcome, is not represented in the PC Monitor, as will explained in the next section.

### Outcome of the process: the European PC Monitor

The final set of indicators included in the PC Monitor resulted from the exclusion procedure as described in the methods section. Sometimes indicators were included after being rephrased. In addition to the many exclusions, a number of new indicators and additional information items have been added resulting from comments made by the evaluators. Before inclusion these new items, their relevance, precision, flexibility and discriminating power were discussed at a consensus meeting with the project partners.

The European PC Monitor describes the structure, process, and outcome of a primary care system by 9 dimensions, 41 features, 99 indicators, and 11 additional information items (see Additional file 1, for a full overview of the PC Monitor). The *structure *of a primary care system is described by its governance, economic conditions, and workforce development. The *process *of a primary care system is described by its access, comprehensiveness, continuity, and coordination of care. The *outcome *of a primary care system is described by its quality of care, and efficiency of care.

Aspects that influence equity in use of primary care services are included in the Monitor. Commonly applied structure and process indicators of inequalities in primary care access and use, have been integrated into several dimensions [8,24]. For example, policy on equality in access (governance), primary care coverage (economic conditions), geographic availability of primary care services (access), and affordability of primary care services (access) are all related to equity.

## Discussion

### Strength and limitations of the indicators

#### Strength

The strength of the PC Monitor is that it builds on well-known frameworks for health care system analysis (such as the structure-process-outcome approach) and primary care research [8,19]. The identified dimensions, features, and indicators are based on the systematic primary care literature review and supported by consensus among primary care experts. Another strength is that in most countries the majority of indicators can be measured by using existing data sources, such as statistics, scientific literature, and policy documents. Some indicators will need an expert opinion for implementation. Furthermore, due to the applied consensus procedure, the Monitor is intended to be applicable to different configurations of primary care (e.g. the different disciplines involved in the provision of primary care).

#### Limitations

The selection and prioritization of dimensions, features and indicators were subject to decisions on several levels. Starting with the search strategy for the systematic literature review, the review process of publications, the data extraction from publications, and finally the evaluation of indicators by the involved primary care experts. Every step of the development process was conducted in agreement with the PHAMEU project partners from ten countries, to safeguard the importance, scientific soundness, and feasibility of the resulting PC Monitor. However, the application of the PC Monitor by the PHAMEU project in the 31 participating countries will ultimately show its feasibility.

The PC Monitor is not exhaustive. Only dimensions marked as important in the systematic literature review are included in the Monitor. Nevertheless, even though the systematic literature review indicated health equity as an important primary care dimension (because primary care can be a means to achieve equity), it was excluded as a dimension in the Monitor because of a lack of health equity indicators that are valid, feasible and measurable, and subject to primary care. However, aspects that influence equity in use of primary care services are included in the Monitor. It is recommended that future research should focus on the development of suitable equity indicators for primary care research.

The reliance on existing data sources is both a strength and a limitation. It can be a limitation because it could reduce the comparability of the resulting primary care information. The comparability would be optimal when data from uniform international surveys are used.

### Application of the PC Monitor

Application of the PC Monitor can be seen as a first test of evaluating what politicians have been 'advertising' about primary care for a while now. The best test of the PC Monitor is to start data collection, as planned in the PHAMEU project. The PC Monitor will be applied in 31 countries by a network of 10 partners. Partners are responsible for data collection in their own and two or three other countries based on their expertise and affinity. Details of the data collection will be tuned to the local situations and availability of sources. For some indicators data can be found in international databases, such as from the OECD, Eurostat, or the WHO Health for All Database. Another source of information are the regularly updated publications in the series 'Health Systems in Transition' (HiT) published by the European Observatory on Health Systems and Policies. Relevant sources can be found via European organisations and networks in primary care (for instance WONCA, EGPRN, EURACT, and EQuiP. Furthermore country information can be found in the international literature. These relatively easy sources will only partly contribute to the data collection for each country. The remainder needs to be found from national sources. As far as national sources can be accessed electronically and in a known language, data can be collected relatively easy by desk research. Websites of national statistical bureaus, professional associations, health inspectorates, educational institutes and national literature databases may be useful. National experts may be needed to get access to grey literature or papers in a foreign language, to help identify sources of missing information, or to deliver 'consensus information'. It is likely that there will be strong heterogeneity of data sources and data. In some countries high quality data for the indicators may be easily available, while in others quality and availability may be low. The network of partners will need to decide about 'softness' criteria for the collected data. If no hard data (e.g. statistics) are available softer data will be applied. For example, in the absence of written sources it may be decided to include consensus among experts. The general principle is to aim for the best available data. This approach is justified as long as the origin of the data is recorded with the data.

It is very likely that not all countries will be able to provide data for each indicator. However, pinpointing gaps in information will also be a valuable result. It will be important that the indicators are evaluated after the PC Monitor has been implemented. This evaluation will result in a final, improved version of the Monitor to be used for future applications.

### Expected impact

Europe-wide application of the PC Monitor is expected to result in up-to-date information on the structure, process and outcome of primary care systems, variation in primary care systems across Europe and knowledge about primary care oriented policy strategies (e.g. related to accessibility or integration). The PC Monitor also offers countries the opportunity to evaluate their primary care system in the context of their policy aims. If the PC Monitor were to be implemented on a structural basis (e.g. every 5 years) it would result in knowledge of trends in primary care.

By creating a basis for routine data collection, the PC Monitor could serve the need of various stakeholder groups for reliable and comparable information. Application of the Monitor will provide European and national decision makers with comprehensive comparisons of primary care policies and models of provision that may enable them to improve the effectiveness of primary care. For the research community, application of the PC Monitor could considerably contribute to the base of evidence and thus advance the state of the art of (primary) health services research. It can also serve future actions in this area, such as health system impact assessments.

## Conclusions

Based on scientific evidence and consensus among experts, an instrument for standardised description and comparison of primary care systems has been developed. Implementation of the instrument in the configurations of primary care in Europe will show the feasibility for producing comparable information. Widespread use of the instrument has the potential to improve the understanding of primary care delivery in different national contexts and thus to create opportunities for better decision making.

## Competing interests

The authors declare that they have no competing interests.

## Authors' contributions

DK and WB drafted each version of the PC Monitor, and DK wrote the manuscript. YB, TC, TH, AH, ML, MO, DRP, IS, PT, AW, AW, and WB evaluated the long list of indicators. All authors reviewed the draft manuscript and read and approved the final manuscript.

## Pre-publication history

The pre-publication history for this paper can be accessed here:

http://www.biomedcentral.com/1471-2296/11/81/prepub

## Supplementary Material

Additional file 1 **The European Primary Care Monitor**. Click here for file
