# Supplementary material for: The european primary care monitor: structure, process and outcome indicators
Source: BMC Fam Pract. 2010 Oct 27;11:81. doi: 10.1186/1471-2296-11-81 (PMC2975652; doi:10.1186/1471-2296-11-81)
Supplement: Additional file 1 — The European Primary Care Monitor. [file 1471-2296-11-81-S1.DOC]

### Additional file 1 – The European Primary Care[[1]](#footnote-2) Monitor

| Dimension: **Governance of the PC system** *(Structure)* | | | |
| --- | --- | --- | --- |
| **Feature** | **Indicator title** | **Indicator** | **Additional information item** |
| GOV1. Primary care goals | Primary care goals | GOV1.1 Have policy documents (by government or important stakeholders) been issued that reflect a clear vision on current and future PC (e.g. for the next 5 years)? [Yes/No] | *GOV1.1a If Yes: In which year? What does this vision entail? What is the status of these documents (e.g. policy paper, law, formal public statement); Which stakeholder?* |
| GOV2. Policy on equality in access | Policy on distribution of human resources | GOV2.1 Is there an explicit governmental policy to regulate the distribution of PC providers and facilities more evenly? [Yes/No] | *GOV2.1a If yes: Please describe the content of these pro-equality measures (e.g. they may be focused on improved working conditions or on obligations for young doctors to work in rural areas?).* |
| GOV3. (De)centralization of PC management & service development | PC within the Ministry of Health | GOV3.1 Does PC has its own department or unit within the Ministry of Health? [Yes/No] | *GOV3.1a Does PC have a budget that can be distinguished from other sectors, such as specialist care? [Yes/No] If yes, please explain at which level this budget is established (e.g. national, regional).* |
|  | PC policy development at regional or local level | GOV3.2 Have responsibilities for PC been decentralized to regional or local level? [Yes/No] | *GOV3.2a If yes, please explain which responsibilities have been decentralized to which levels (for instance, setting priorities; aspects of service provision).* |
|  | Stakeholder involvement in PC policy development | GOV3.3 Do organisations of stakeholders contribute to PC policy development? (e.g. health insurers, medical professionals, or representatives of patients or consumers). [Yes/No] | *GOV3.3a If yes, please explain in which way they contribute to PC policy development (e.g. in regular formal consultations or incidentally and informal).* |
|  | (De)centralization of PC service delivery | GOV3.4 Has community influence on the provision of PC services been organised on a national or regional level? [not applicable, it is not used/ yes, on a national level/ yes in some regions/ yes, incidentally at local level]. | *GOV3.4a If yes, which of the following forms apply:  1. Via ownership of PC facilities by authorities:  a. State; b. Region; c. Local.  2. (Voluntary) patient councils with PC facilities. 3. Local/regional/national PC satisfaction surveys; 4. Volunteer work in PC facilities; 5. Other [Please fill in]…..* |
| GOV4. PC Quality Management Infrastructure | Coordination of quality management | GOV4.1 If state inspection on health care exists, does it have a specific unit for PC? [Yes/No/Not applicable] | - |
|  | Certification of providers | GOV4.2 Do formal requirements exist for physicians (such as GPs/Family doctors) to work in PC? [Yes/No] | *GOV4.2a If yes, what are the obligatory professional requirements for physicians to practice in PC? (e.g. Having completed postgraduate specialisation or obligatory CME). Please specify for GPs/FDs and possible other specialists working in PC* |

| Dimension: **Governance of the PC system** *(Structure)* | | | |
| --- | --- | --- | --- |
| **Feature** | **Indicator title** | **Indicator** | **Additional information item** |
|  | Licensing of facilities | GOV4.3 Do formal requirements exist for PC practices or facilities to operate? [Yes/No] | *GOV4.3a If yes, What are the requirements for PC practices or facilities to operate?* |
|  |  |  | *GOV4.3b Please mention important voluntary mechanisms to maintain and improve the quality of care. (e.g. clinical guidelines, voluntary peer-review mechanisms).* |
|  | Development of clinical guidelines | GOV4.4 Have evidence based clinical guidelines been produced for specific use by GPs? [Yes/No] | *GOV4.4a If yes: What is the usual mode of production of these guidelines?*  *1. Issued by a national agency such as the Ministry of Health [yes / no];*  *2. Issued by a college or association of GPs [yes / no];*  *3. Adapted foreign guidelines [yes / no];*  *4. Developed by medical specialists [yes / no] ;*  *5. Other:..….[fill in]* |
| GOV5. Patient advocacy | Patient rights | GOV5.1 Have any laws/regulation pertaining to the following patients’ rights in PC been implemented?  1. Informed consent [Yes/No]*;* 2. Patient access to own medical files [Yes/No]*;* 3. Confidential use of medical records [Yes/No]*;* 4. Availability of a procedure to process patient complaints in PC facilities [Yes/No]*.* | - |
| GOV6. Multidisciplinary collaboration | Multidisciplinary collaboration | GOV6.1 Has a governmental policy on cooperation or integration of PC services been laid down in a law or policy paper? [Yes/No/ Not applicable, because no such policy exists] | *GOV6.1a If yes, what is the core of this policy and which PC providers are targeted?* |

| Dimension: **Economic conditions of the PC system** *(Structure)* | | | |
| --- | --- | --- | --- |
| **Feature** | **Indicator title** | **Indicator** | **Additional information item** |
| ECO1. Primary care expenditure | Total PC expenditure | ECO1.1 Total expenditure on PC as % of total expenditure on health | *-* |
|  | Expenditure on prevention and public health | ECO1.2 Total expenditure on prevention and public health as % of total expenditure on health. | *-* |
| ECO2. Primary care coverage | Total PC coverage | ECO2.1 % of the population fully covered or insured for PC costs. | *-* |
|  | GP services coverage | ECO2.2 % of the population covered or insured for costs of GP services (office and at home) . | ECO2.2a If copayment applies, please explain the volume of copayment |
|  | Medicines coverage | ECO2.3 % of the population covered or insured for medicines prescribed in primary care / GP. | ECO2.3a If copayment applies, please explain the volume of copayment |
|  | Uninsured population | ECO2.4 % of the population uninsured for medical expenses (this may be an estimation) | *-* |
|  | Out-patient medical care coverage by social insurance | ECO2.5 Social health insurance coverage for out-patient medical care by % of population. | *-* |
| ECO3. Employment status of PC workforce | Employment status of GPs | ECO3.1 % of GPs that are:  1a. Salaried with national, regional or local authorities; 1b. Salaried with other physicians; 2a. Self-employed with contract to health insurance fund(s) or health authority; 2b. Self-employed without contract (paid by patients out-of-pocket).3. Other mode | *-* |
| ECO4. Remuneration system of PC workforce | Remuneration system for salaried GPs | ECO4.1 How are salaried GPs paid? 1. Flat salary; 2. salary related to the number of their patients; 3 Salary related to both the number of their patients and indicators of performance | *ECO4.1a If they receive a performance-related salary: please explain which elements are taken into account.* |
|  | Remuneration system for self-employed GPs | ECO4.2 How are self-employed GPs paid? 1. Fee-for-service payment; 2. Capitation payment; 3. Mix of capitation and fee-for-service payment. 4. Mix of capitation and fee-for-service and other specific components (e.g. bonus for working in disadvantaged areas etc.). | *ECO4.2a If they receive a payment consisting of other components than capitation or fee-for service, please explain to what targets or situations these are related.* |
| ECO5. Income of PC workforce | Income of GPs | ECO5.1 What is the (estimated) gross annual income (in Euros) of a ‘mid-career’ GP (about 10 years experience and with an average size of practice)? | *ECO5.1a Does this income include costs for running the practice (premises; equipment; care; employed staff)?* |

| Dimension: **PC Workforce Development** *(Structure)* | | | |
| --- | --- | --- | --- |
| **Feature** | **Indicator title** | **Indicator** | **Additional information item** |
| WFD1. Profile of PC workforce | Type of PC professionals | WFD1.1 To which of the following medical, para-medical and nursing disciplines people have direct access (which means without referral or intervention by another medical provider)?  Please, indicate on the list and add disciplines if applicable. Also indicate with each discipline whether they exclusively work in PC or also provide services on referral (for instance in another setting, such as a hospital)   - GP /Family physician - Gynaecologist/obstetrician - Paediatrician - Specialist of Internal medicine - Ophthalmologist - ENT specialist - Cardiologist Neurologist - Surgeon - Primary care / GP practice nurse - Specialised nurse (eg. on diabetes) - Home care nurse - Physiotherapists (ambulatory) - Midwife (ambulatory) - Occupational therapist - Speech therapist - Dentist - Other:, namely: ……………….   ……………… | *-* |
|  | Age distribution GPs | WFD1.2 Average age of practicing GPs. | *WFD1.2a What is the age distribution among practicing GPs? Please fill in the % of GPs that are: <35 years of age; 35-45 years of age; 45-55 years of age; 55+ years of age.* |
|  | Workload GPs | WFD1.3 Average number of working hours per week of GPs (including: hours for keeping up to date and for administration; excluding: hours on call (in evening, weekends etc.) | *-* |
| WFD2. Status & Responsibilities of PC disciplines | Recognition / responsibilities of GPs | WFD2.1 Have tasks/duties of GPs or family doctors been described in a law or policy document? [Yes/No] | *WFD2.1a If yes, please fill in the name of the documents, who issued it, and year of issue.* |

| Dimension: **PC Workforce Development** *(Structure)* | | | |
| --- | --- | --- | --- |
| **Feature** | **Indicator title** | **Indicator** | **Additional information item** |
|  | Financial status of GPs compared to a specialist | WFD2.2 How does the gross annual income (in Euros) of a mid-career GP (about 10 yrs experience with average size of practice) relate to the gross annual income of the following medical, para-medical and nursing disciplines of the same age? Please give an estimation whether a GP’s income is  [Much lower / lower / equal / higher / much higher].   - Gynaecologist/obstetrician - Paediatrician - Specialist of Internal medicine - Ophthalmologist - ENT specialist - Cardiologist - Neurologist - Surgeon - Primary care / GP practice nurse - Specialised nurse (eg. on diabetes) - Home care nurse - Physiotherapists (ambulatory) - Midwife (ambulatory) - Occupational therapist - Speech therapist - Dentist | *-* |
|  | Attractiveness of FM among medical students | WFD2.3 What % of all medical graduates chooses to enrol in postgraduate training in family medicine (within 1 year after graduation)? (use the most recent available year, and fill this in) […%, with reference year:..) | *-* |
| WFD3. PC Workforce supply and planning | Development of workforce supply | WFD3.1 Please indicate the % by which the supply (total number) of directly accessible medical, para-medical and nursing disciplines has increased [+….%] or reduced [-….%] over the most recent available 5 year period. Please also indicate the years applied [Years ….-….].   - GP /Family physician - Gynaecologist/obstetrician - Paediatrician - Specialist of Internal medicine - Ophthalmologist - ENT specialist - Cardiologist - Neurologist - Surgeon - Primary care / GP practice nurse - Specialised nurse (eg. on diabetes) - Home care nurse - Physiotherapists (ambulatory) - Midwife (ambulatory) - Occupational therapist - Speech therapist - Dentist - Other:, namely: ………………. | *-* |

| Dimension: **PC Workforce Development** *(Structure)* | | | |
| --- | --- | --- | --- |
| **Feature** | **Indicator title** | **Indicator** | **Additional information item** |
|  | GP-Specialist ratio | WFD3.2 Total nr. of active GPs as a ratio to total nr. of active specialists. | *-* |
|  | Workforce planning | WFD3.3 Are data available from studies on PC workforce capacity needs and development in the future? [Yes/No] | *WFD3.3a If yes, for which PC disciplines and what was the latest date of publication?* |
| WFD4. Academic status of PC | Academic status of FM/ general practice | WFD4.1% of medical universities (or universities with a medical faculty) with a postgraduate programme in family medicine. | *WFD4.1a In what year was postgraduate training in family medicine first introduced?* |
|  |  |  | *WFD4.1b How many departments of family medicine are there at medical universities (or universities with medical faculties) in this country?* |
|  | FM/ general practice education | WFD4.2 Is family medicine subject in the undergraduate medical curriculum? [Yes/No] | *WFD4.2a What is the duration of a postgraduate programme in family medicine in this country, and how many months do trainees spend in a PC setting?* |
|  | Education of nurses in PC | WFD4.3 Is there professional training specifically for:   - district- or community nurses? [Yes/No] - PC/GP practice nurses [Yes/No] | *WFD4.3a If yes, what is its duration?* |
| WFD5. Medical associations | Professional association of GPs | WFD5.1 Do national associations or colleges of GPs exist in this country? [Yes/No] | *WFD5.1a If yes, please provide the name(s), number of GPs being a member, and indicate which of the following activities the association/organization undertakes: 1. Defending financial/material interests; 2. Professional development (e.g. guideline development); 3. Education; 4. Scientific activities.* |
|  | Professional Journal on GP | WFD5.2 Is a journal on family medicine/general practice being published in this country? [Yes/No] | *WFD5.2a Please provide its name, number of issues per year, and the number of subscriptions.*  *Also indicate for each journal a characterisation of its content [primarily; about 50/50; minor importance]:*  *News; opinions; popular articles; scientific articles (peer reviewed; with abstract in English)* |
|  | Professional association of PC nurses | WFD5.3 Do national associations or organisations of PC nurses exist in this country? [Yes/No] | *WFD5.3a If yes, please provide the name(s), number of nurses being a member, and indicate which of the following activities the association/organization undertakes: 1. Defending financial/material interests; 2. Professional development (e.g. guideline development); 3. Education; 4. Scientific activities.* |
|  | Professional Journal on PC nursing | WFD5.4 Is a professional journal on PC nursing being published in this country? [Yes/No] | *WFD5.4a Please provide its name, number of issues per year, and the number of subscriptions.* |

| Dimension: **Access to PC services** *(Process)* | | | |
| --- | --- | --- | --- |
| **Feature** | **Indicator title** | **Indicator** | **Additional information item** |
| ACC1. National Availability of PC services | Density available PC workforce | ACC1.1 Please provide the total number of directly accessible medical, para-medical and nursing disciplines available per 100,000 population:   - GP /Family physician: ……. - Gynaecologist/obstetrician: ……. - Paediatrician: ……. - Specialist of Internal medicine: ……. - Ophthalmologist: ……. - ENT specialist: ……. - Cardiologist: ……. Neurologist: ……. - Surgeon: ……. - Primary care / GP practice nurse: ……. - Specialised nurse (eg. on diabetes) : ……. - Home care nurse: ……. - Physiotherapists (ambulatory) : ……. - Midwife (ambulatory) : ……. - Occupational therapist: ……. - Speech therapist: ……. - Dentist: ……. - Other:, namely: ……………….: …….   ………………: ……. | *-* |
| ACC2. Geographic availability of PC services | Availability of GPs by region | ACC2.1 Difference between region, province or state with highest and with lowest density of GPs (per 100,000 population). | *ACC2.1a Availability of GPs by region, province or state per 100,000 population.* |
|  | Urban-Rural availability of GPs | ACC2.2 Difference between average urban density of GPs (per 100,000 population) and average rural density of GPs. | *-* |
|  | Shortage of GPs | ACC2.3 Do (regional or national) shortages exist of GPs according to usual national norms? [No shortage / Shortage in some regions / Modest shortage nation wide / Severe shortage nation wide/ Not applicable, because no norms exist] | *-* |
|  | Shortage of community pharmacists | ACC2.4 Do problems exist in the availability of medicines in rural areas du to lack of pharmacies? [Yes/No] | *-* |
| ACC3. Accommodation of accessibility | Opening hours | ACC3.1 Are GP practices or PC centres obliged to have a minimum number of opening hours or days? | *ACC3.1a If yes, how many hours or days?* |

| Dimension: **Access to PC services** *(Process)* | | | |
| --- | --- | --- | --- |
| **Feature** | **Indicator title** | **Indicator** | **Additional information item** |
|  | Home visits | ACC3.2 Average nr. of home visits per week per GP. | *-* |
|  | Organizational access arrangements | ACC3.3 To what extent do the following organizational arrangements commonly exist in GP practices or PC centres? [(almost) always present / usually present / occasionally present / seldom or never present]:  1. Telephone consultations; 2. E-mail consultations; 3. Practices having a website; 4. Offering special sessions or clinics for certain patient groups (e.g. diabetics, pregnant women, hypertensive patients etc) 5. Appointment systems for the majority of the patient contacts. | *-* |
|  | After-hours PC | ACC3.4 To what extent are the following models for the provision of after-hours PC commonly used? [(almost) always used / usually used / occasionally used / seldom or never used]:  1) Practice-based services: GPs within one practice or organized in a group of practices look after their patients on out-of-hours schedules;  2) PC cooperatives: GPs in a region from several groups, supported by additional personnel, provide after-hours PC mostly in non-profit, large-scale organizations, which include telephone triage and advice, office for face-to-face contact, and house calls.  3) Deputizing services (outsourcing): companies employing doctors take over the provision of after-hours care;  4) Hospital emergency departments provide PC by taking care of health problems after office hours;  5) After-hours PC centres: These are (walk-in) centres for face-to-face contact with a GP or nurse;  6) Other out-of-hours PC/GP service schemes in place. | *ACC3.4a Please explain if this scheme has been implemented uniformly all over the country or do significant regional differences exist? If ‘other schemes’ are in place shortly explain services and providers.* |
| ACC4. Affordability of PC services | Cost-sharing for GP care | ACC4.1 Do patients normally need to pay for [no payment / some payment / payment of the full amount]: 1. A visit to their GP; 2. Medicines or injections prescribed by their GP; 3. A visit to a specialist prescribed by their GP; 4. A visit of their GP at the patient’s home. | *ACC4.1a Please explain if exemptions exist for certain groups of patients (which groups; for which services).* |
|  | Patient dissatisfaction with PC prices | ACC4.2 % of patients that rate GP care as not very or not at all affordable. | - |
| ACC5. Acceptability of PC services | Patient satisfaction with access of PC in general | ACC5.1 % Patients that find it easy to reach and gain access to GPs. | - |

| Dimension: **Continuity of PC services** *(Process)* | | | |
| --- | --- | --- | --- |
| **Feature** | **Indicator title** | **Indicator** | **Additional information item** |
| CON1. Longitudinal continuity of care | Patient list system | CON1.1 Do GPs have a patient list system? [Yes/No] | *CON1.1a Average population size per GP.* |
|  | Stability of Patient-Provider relationship | CON1.2 % of patients reporting to visit their usual PC provider for their common health problems. | *-* |
| CON2. Informational continuity of care | Medical record keeping | CON2.1 % of GPs keeping (or reporting to keep) clinical records for all patient contacts routinely. | ***-*** |
|  | Electronic clinical support systems | CON2.2 To what extent do GPs have a computer at their disposal in their office? [(almost) always / usually / occasionally / seldom or never]. | *CON2.2a For which of the following purposes are GPs usually using a computer in their practice?: [answer options per category: yes/no]*  *1.Booking appointments with patients; 2.Writing bills/financial administration; 3. Prescription of medicines; 4. Keeping medical records of patients; 5. Searching expert information on the internet; 6. Communicating patient information to specialists; 7. Communicating prescriptions to pharmacists.* |
|  |  |  | *CON2.2b Are clinical record systems in PC/GP able to generate lists of patients by diagnosis or health risk? (e.g. patients with asthma or diabetes, or smokers)* |
|  | Referral system | CON2.3 To what extent are GPs using referral letters (including relevant information on diagnostics and treatment performed) when they refer to a medical specialist? [(almost) always / usually / occasionally / seldom or never]. | *-* |
|  | Incoming clinical information procedures | CON2.4 Do PC practices receive information within 24 hours about contacts that patients have with out of hours services? [(almost) always / usually / occasionally / seldom or never]. | *-* |
|  | Specialist-GP communication | CON2.5 To what extent do specialists communicate back to referring GP after an episode of treatment? [(almost) always / usually / occasionally / seldom or never]. | *-* |

| Dimension: **Continuity of PC services** *(Process)* | | | |
| --- | --- | --- | --- |
| **Feature** | **Indicator title** | **Indicator** | **Additional information item** |
| CON3. Relational continuity of care | Physician choice | CON3.1 Are patients free to choose the PC centre and GP they want to register with?  [Yes, patients can freely choose any centre or GP / Patients are free to choose a centre, but they are assigned to a GP in that centre / Patients are assigned to a centre in their area, but they are free to register with any GP in that centre / No, patients are assigned to a PC centre in their area, and they are assigned to a GP in that centre]. | *CON3.1a Please explain if in reality the situation is not as it is intended to e (except for the usual limited choice in rural areas).* |
|  | Patient satisfaction | CON3.2 % of patients who are satisfied with:   - their relation with their GP/PC physician - with the available time during consultations with their GP/PC physician - their trust in their GP/PC physician - the explanation their GP or PC physician gives of problems, procedures and treatments | *-* |

| Dimension: **Coordination of PC services** *(Process)* | | | |
| --- | --- | --- | --- |
| **Feature** | **Indicator title** | **Indicator** | **Additional information item** |
| COO1. Gatekeeping system | Gatekeeping system | COO1.1 Do patients need a referral to access the following medical, para-medical and nursing disciplines?  [1. Yes, a referral is normally required 2. No they have direct access 3. Direct access is possible if costs of the visit are paid privately (out of pocket or refunded from a complementary insurance)]   - Gynaecologist/obstetrician - Paediatrician - Specialist of Internal medicine - Ophthalmologist - ENT specialist - Cardiologist - Neurologist - Surgeon - Primary care / GP practice nurse - Specialised nurse (eg. on diabetes) - Home care nurse - Physiotherapists (ambulatory) - Midwife (ambulatory) - Occupational therapist - Speech therapist - Dentist | - |
| COO2. Skill-mix of PC providers | Shared practice | COO2.1 % of PC practices that are:  - Single handed (solo);  - 2 or 3 GPs in the same building without medical specialists;  - 4 or more GPs in the same building without medical specialists;  - Mixed practice with GPs and medical specialists. | - |
|  | Cooperation within PC | COO2.2 Is it common for GPs to have regular face-to-face meetings (at least once per month) with the following professionals? [Yes, it often occurs / Yes, it usually occurs / No, it occasionally occurs / It seldom or never occurs]. Please explain.   - Other GP(s) - Practice nurse(s) - Nurse practitioner(s) - Home care nurse(s) - Midwife/birth assistant(s) - PC physiotherapist(s) - Community pharmacist(s) - Social worker(s) - Community mental health workers | - |

| Dimension: **Coordination of PC services** *(Process)* | | | |
| --- | --- | --- | --- |
| **Feature** | **Indicator title** | **Indicator** | **Additional information item** |
|  | Substitution | COO2.3 How usual are the following modes of care by nurses in PC/GP? [very common / usual/ rare/ uncommon]   1. Nurse-led diabetes clinics in PC/GP 2. Nurse-led health education (e.g. for stop smoking or pregnant women) | **-** |
| COO3. Collaboration of PC-Secondary care | Specialist outreach | COO3.1 How common are the following forms of cooperation between GP/PC and medical specialists? [very common / usual/ rare/ uncommon]  1. Medical specialists visiting a PC practice to provide specialist care normally provided in hospital (replaced specialist care).  2. Medical specialists visiting a PC practice to provide joint care with a GP (joint consultations).  3. Clinical lessons by a medical specialist for GPs. | *COO3.1a How common is it that GPs ask (telephone) advice from the following medical specialists?[very common / usual/ rare/ uncommon]*  *1. Paediatricians; 2. Internists; 3. Gynaecologists; -Surgeons; 4.Neurologists; 5. Dermatologists; 6. Geriatrists.* |
| COO4. Integration of public health in PC | Epidemiologic al data set | COO4.1 Are clinical patient records from GP/PC used at regional or local level to identify health needs or priorities for health policy? [routinely (health statistics) / incidentally / seldom or never used] | - |
|  | Community health surveys | COO4.2 Are community health surveys conducted to improve the quality and responsiveness of PC? [Regularly nationwide / incidentally nationwide / regularly at local or regional level / incidentally at local or regional level] | - |

| Dimension: **Comprehensiveness of PC services** *(Process)* | | | |
| --- | --- | --- | --- |
| **Feature** | **Indicator title** | **Indicator** | **Additional information item** |
| COM1. Medical equipment available | Medical equipment available | COM1.1 How common is it that PC facilities have the following equipment available at the premises: [(almost) always available / usually available / occasionally available / seldom or never available]  1. infant scales; 2. glucose tests; 3. dressings/bandages; 4. otoscope; 5.ECG; 6. urine strips; 7. instruments for stitching wounds; 8. gynaecological speculum; 9. peak flow meter | *-* |
| COM2. First contact for common health problems | First contact care | COM2.1 To what extent will patients with the following health problems visit a GP for first contact care? [(almost) always / usually / occasionally / seldom or never].   - child with severe cough - child aged 8 with hearing problem - woman aged 18 asking for oral contraception - woman aged 20 for confirmation of pregnancy - woman aged 35 with irregular menstruation - woman aged 35 with psychosocial problems - woman aged 50 with a lump in her breast - man aged 28 with a first convulsion - man with suicidal inclinations - man aged 52 with alcohol addiction problems | *COM2.1a Please indicate for each health problem to which other specialty(ies) (other than a GP) these patients may (also) address for first the contact? (please list 1 or 2, if applicable)*   - child with severe cough - child aged 8 with hearing problem - woman aged 18 asking for oral contraception - woman aged 20 for confirmation of pregnancy - woman aged 35 with irregular menstruation - woman aged 35 with psychosocial problems - woman aged 50 with a lump in her breast - man aged 28 with a first convulsion - man with suicidal inclinations - man aged 52 with alcohol addiction problems |
| COM3. Treatment and follow-up of diseases | Treatment and follow-up of diseases | COM3.1 To what extent will patients with the following diseases receive treatment / follow up care from their GP? [(almost) always / usually / occasionally / seldom or never].   - Chronic bronchitis - Peptic ulcer - Congestive heart failure - Pneumonia - Uncomplicated diabetes type II - Rheumatoid arthritis - Mild depression - Cancer (in need for palliative care) - Patients admitted to a nursing home/convalescent home | *COM3.1a Which specialties (besides a GP) are (also) treating in the below mentioned cases? (please list 1 or 2, if applicable)*   - Chronic bronchitis - Peptic ulcer - Congestive heart failure - Pneumonia - Uncomplicated diabetes type II - Rheumatoid arthritis - Mild depression - Cancer (in need for palliative care) - Patients admitted to a nursing home/convalescent home |
|  | GP contacts without referral | COM3.2 % of total patient contacts handled solely by GPs without referrals to other providers. | *-* |

| Dimension: **Comprehensiveness of PC services** *(Process)* | | | |
| --- | --- | --- | --- |
| **Feature** | **Indicator title** | **Indicator** | **Additional information item** |
| COM4. Medical technical procedures | Medical technical procedures | COM4.1To what extent do GPs or GP/PC practice nurses carry out the following activities if one of their patients would need so? [(almost) always / usually / occasionally / seldom or never].   - Wedge resection of ingrown toenail - Removal of sebaceous cyst from hairy scalp - Wound suturing - Excision of warts - Insertion of IUD - Removal of rusty spot from the cornea - Fundoscopy - Joint injection - Strapping an ankle - Setting up an intravenous infusion | *COM4.1a Which specialties (besides GPs or* GP/PC practice nurses) would *(also) provide the procedure? (please list 1 or 2, if applicable)*   - Wedge resection of ingrown toenail - Removal of sebaceous cyst from hairy scalp - Wound suturing - Excision of warts - Insertion of IUD - Removal of rusty spot from the cornea - Fundoscopy - Joint injection - Strapping an ankle - Setting up an intravenous infusion |
| COM5. Preventive care | Preventive care | COM5.1 To what extent do GPs carry the following preventive activities? [(almost) always / usually / occasionally / seldom or never].   - Immunization for tetanus - Allergy vaccinations - Testing for sexually transmitted diseases - Screening for HIV/AIDS - Influenza vaccination for high-risk groups - Cervical cancer screening - Breast cancer screening - Cholesterol level checking | *COM5.1a Which specialties (besides GPs*) would *(also) provide the preventive activity? (please list 1 or 2, if applicable)*   - Immunization for tetanus - Allergy vaccinations - Testing for sexually transmitted diseases - Screening for HIV/AIDS - Influenza vaccination for high-risk groups - Cervical cancer screening - Breast cancer screening - Cholesterol level checking |
| COM6. Mother and child & Reproductive health care | Mother and child & Reproductive health care | COM6.1 To what extent do GPs provide the following health services to their patients who need so? [(almost) always / usually / occasionally / seldom or never].   - Family planning / contraceptive care - Routine antenatal care (in line with national scheme) - Routine paediatric surveillance to children up to 4 years | *COM6.1a If not the GP, which other specialty(ies) would provide this health service? (please list 1 or 2, if applicable)*   - Family planning / contraceptive care - Routine antenatal care (in line with national scheme) - Routine paediatric surveillance to children up to 4 years |
|  |  | COM6.2 To what extent are GPs (or practice nurses) involved in infant vaccination on: [(almost) always / usually / occasionally / seldom or never].   - diphtheria - tetanus - pertussis - measles - hepatitis B - mumps - rubella | *COM6.2a If not the GP or practice nurse, which other specialty(ies) would provide this health service? (please list 1 or 2, if applicable)*   - diphtheria - tetanus - pertussis - measles - hepatitis B - mumps - rubella |

| Dimension: **Comprehensiveness of PC services** *(Process)* | | | |
| --- | --- | --- | --- |
| **Feature** | **Indicator title** | **Indicator** | **Additional information item** |
| COM7. Health promotion | Health promotion | COM7.1 To what extent do GPs provide the following individual counselling if this would be needed in the practice population? [(almost) always / usually / occasionally / seldom or never].   - Counselling in case of obesity - Counselling in case of poor physical activity - Counselling in case of smoking cessation - Counselling in case of problematic alcohol consumption | *COM7.1a If not the GP, which other specialty(ies) would provide this counselling? (please list 1 or 2, if applicable)*   - Counselling in case of obesity - Counselling in case of poor physical activity - Counselling in case of smoking cessation - Counselling in case of problematic alcohol consumption |
|  | Health education (groupwise) | COM7.2 To what extent are GPs (alone or with others) involved in groupwise health education to their patients (on topics like healthy diet; physical activity; smoking; use of alcohol etc).? [usual task of GPs / incidental task / rarely or never provided by GPs] | *COM7.2a If not the GP, which other specialty(ies) would provide this groupwise health education? (please list 1 or 2, if applicable* |

| Dimension: **Quality of PC** *(Outcome)* | | | |
| --- | --- | --- | --- |
| **Feature** | **Indicator title** | **Indicator** | **Additional information item** |
| QUA1. Prescribing behaviour of PC providers | Annual prescriptions | QUA1.1 The average number of prescriptions annually provided by GPs per 1000 contacts and/or per 1000 registered patients. (Please use latest available data, and indicate the year). | *-* |
|  | Antibiotics consumption | QUA1.2 The defined daily doses of antibiotics use in ambulatory care per 1000 inhabitants per day | *-* |
| QUA2. Quality of diagnosis and treatment in PC | Avoidable hospitalization | QUA2.1 The number of hospital admissions for people with the following conditions per 100.000 population per year. Please use the latest available data, and indicate the year.   - diagnosis of dehydration/gastroenteritis *(ICD-10 codes: E86, K52.2, K52.8, K52.9)* - diagnosis of kidney *infection (ICD-10 codes: N10, N11, N12, N13.6)* - diagnosis of perforated ulcer *(ICD-10 codes: K25.0–K25.2, K25.4–K25.6, K26.0–K26.2, K26.4–K26.6, K27.0–K27.2, K27.4–K27.6, K280–282, K284–K286)* - diagnosis of pelvic inflammatory disease *(ICD-10 codes: N70, N73, N74)*   a diagnosis of ear, nose and throat (ENT) infections *(ICD-10 codes: H66, H67, J02, J03, J06, J31.2)* | - |
| QUA3. Quality chronic diseases management | Diabetes care | QUA3.1 Crude percentage of the diabetic population aged >25 with cholesterol 5>mmol/ll (crude percentage; use latest available year; please indicate the year) | - |
|  |  | QUA3.2 Crude percentage of diabetic population aged >25 years with blood pressure above 140/90 mm Hg measured in the last 12 months.(crude percentage; use latest available year; please indicate the year) | - |
|  |  | QUA3.3 Crude percentage of diabetic population aged >25 years with HbA1C > 7,0%. (crude percentage; use latest available year; please indicate the year) | - |
|  |  | QUA3.4 Crude percentage of diabetic population aged >25 years with overweight and obesity and BMI measured in the last 12 months. (crude percentage; use latest available year; please indicate the year) | - |
|  |  | QUA3.5 Crude percentage of diabetic population aged >25 years with eye fundus inspection in the last 12 months (crude percentage; use latest available year; please indicate the year) | *-* |

| Dimension: **Quality of PC** *(Outcome)* | | | |
| --- | --- | --- | --- |
| **Feature** | **Indicator title** | **Indicator** | **Additional information item** |
|  | COPD care | QUA3.6 Percentage of individuals with COPD which have ever had a lung function measurement during the last year. (use latest available year; please indicate the year) | *-* |
|  |  | QUA3.7 Percentage of individuals with COPD that have had a follow-up visit in primary care during the last year. (use latest available year; please indicate the year) | *-* |
|  | Asthma care | QUA3.8 Percentage of individuals with wheeze in the last 12 months or diagnosed with asthma which have had a lung function measurement during the last year. (use latest available year; please indicate the year) | *-* |
|  |  | QUA3.9 Proportion of individuals having had wheeze  in the last twelve months with a diagnosis of asthma that have had a follow-up visit in primary care during the last year. (use latest available year; please indicate the year) | *-* |
|  |  | QUA3.10 The number of hospital admissions for people with a diagnosis of asthma per 100.000 population per year (in the most recent year-please indicate the year). (use latest available year; please indicate the year) | *-* |
| QUA4. Quality of Maternal and Child health care | Infant vaccination | QUA4.1 % of infants vaccinated within PC against: [% or not applicable because outside PC]   - diphteria. - tetanus. - pertussis - measles. - hepatitis B. - mumps - rubella | - |
| QUA5. Quality of preventive care | Vaccine preventable ambulatory care sensitive condition | QUA5.1 % population aged 60+ vaccinated against flu. [% or not applicable because outside PC] | *-* |
|  | Breast cancer screening | QUA5.2 % of women aged 52-69 yrs who had at least 1 mammogram in the past 3 yrs. [% or not applicable because outside PC] | *-* |
|  | Cervical cancer screening | QUA5.3 % of women aged 21-64 yrs who had at least 1 Pap test in the past 3 yrs. [% or not applicable because outside PC] | - |

| Dimension: **Efficiency of PC** *(Outcome)* | | | |
| --- | --- | --- | --- |
| **Feature** | **Indicator title** | **Indicator** | **Additional information item** |
| EFF1. General practice efficiency | Home visits | EFF1.1 Nr. of home visits as % of all GP-patient contacts. (use latest available year; please indicate the year) | - |
|  | Telephone consultations | EFF1.2 Nr. of telephone consultations as % of all GP-patient contacts. (use latest available year; please indicate the year) | - |
|  | Duration of GP consultation | EFF1.3 Average consultation length (in minutes) of GPs. (use latest available year; please indicate the year) | - |
|  | GP consultations | EFF1.4 Number of GP consultations per capita per year (use latest available year; please indicate the year) | - |
|  | Referrals to medical specialists | EFF1.5 Number of new referrals from GPs to medical specialists per 1000 listed patients per year. (use latest available year; please indicate the year) | - |

1. Primary care is defined as the first level of professional care where people present their health problems and where the majority of the population’s curative and preventive health needs are satisfied. [↑](#footnote-ref-2)
